# Long-Term Biocompatibility of a Highly Viscously Thiol-Modified Cross-Linked Hyaluronate as a Novel Vitreous Body Substitute

**DOI:** 10.3389/fphar.2022.817353

**Published:** 2022-03-02

**Authors:** Jose Hurst, Annekatrin Rickmann, Nele Heider, Christine Hohenadl, Charlotte Reither, Andreas Schatz, Sven Schnichels, Kai Januschowski, Martin S. Spitzer

**Affiliations:** ^1^ Centre of Ophthalmology, University Eye Hospital Tübingen, Tübingen, Germany; ^2^ Eye Clinic Sulzbach, Knappschafts Hospital Sulzbach/Saar, Sulzbach, Germany; ^3^ Croma Pharma GmbH, Leobendorf, Austria; ^4^ Mount St. Peter Eye Clinic Trier, Trier, Germany; ^5^ Department of Ophthalmology, University Medical Center Hamburg-Eppendorf (UKE), Hamburg, Germany

**Keywords:** vitreoretinal surgery, hyaluronic acid, thiol-modified, crosslinked, vitreous substitute, artificial vitreous

## Abstract

**Purpose:** In surgical ophthalmology, the treatment of complicated retinal and vitreous diseases is one of the central challenges. For this purpose, the vitreous body is removed as part of the standard therapy and replaced by a temporary tamponade to stabilize the position of the retina. Since the tamponading properties of previous materials such as silicone oils, gases, or semi-fluorinated alkanes are a combination of their surface tension and their buoyancy vector, they cannot completely fill the vitreous cavity. The aim of this work was to test *in vivo* a novel vitreous body substitute (ViBos strong) based on cross-linked hyaluronic acid for its compatibility.

**Methods:** A pars plana vitrectomy with posterior vitreous detachment was performed in the right eye of 18 pigmented rabbits, with subsequent injection of ViBos strong. Follow-up examination included slit-lamp examination, funduscopy, intraocular pressure measurements (IOP), optical coherence tomography (OCT), and electroretinogram (ERG) measurements. The rabbits were sacrificed at three different time points (1, 3, and 6 months; each 6 animals) and examined macroscopically and prepared for histological examination (HE staining) and immunohistochemistry (Brn3a and glial fibrillary acidic protein (GFAP)).

**Results:** ViBos strong demonstrated good intraoperative handling and remained stable for at least 1 month and degraded slowly over 6 months. IOP was within clinical acceptable values at all follow-up examinations. Retinal function was well preserved after instillation of the hydrogel and comparable to the untreated eye after 6 months in OCT, ERG, and histological examinations. An increase in the GFAP expression was found in the surgery eyes, with a peak in the 3-month group. The Brn3a expression was not significantly affected by vitrectomy with ViBos strong.

**Conclusion:** Highly viscously thiol-modified cross-linked hyaluronate showed a good biocompatibility in rabbit eyes over 6 months after vitrectomy, making it a promising potential as a vitreous substitute.

## Introduction

In the treatment of pathologies affecting the posterior pole, the vitreous body needs to be removed by vitrectomy following stabilization of the retina with an endotamponade, like silicone oils or gases. Current clinically used endotamponades are effective, especially in promoting retinal reattachment, but are disadvantageous due to their hydrophobic nature, refractive index, and density compared to the human vitreous, resulting in immediate blurred vision after surgery and complications such as raised intraocular pressure (IOP), prolonged inflammation, emulsification, or cataract formation (Kim et al., 2008; Duan et al., 2011; Lin et al., 2021; Schulz et al., 2021). The basic reason for the limitation of endotamponades is their hydrophobic character in a hydrophilic environment of the vitreous cavity and acting *via* the two physical functions “buoyancy vector” and “surface/interfacial tension.” The tamponade vector can only act in one direction, so complete filling of the vitreous cavity is not possible (Schnichels et al., 2017; Szurman, 2017). Furthermore, while gas endotamponades diffuse out over time, silicone oils are nonbiodegradable and must be surgically removed (Duan et al., 2011; Lin et al., 2021).

Therefore, there is great demand to develop an ideal vitreous endotamponade that mimics the physiological properties of a natural vitreous body to improve therapeutic efficacy and safety. Recent tamponade strategies aim at hydrophilic, polymeric hydrogel-based systems due to their favorable physical and mechanical properties such as high water content, high optical transparency, suitable refractive indices, viscosity, swelling pressure, adjustable rheological properties, and adaptable biocompatibility (Schulz et al., 2020; Lin et al., 2021; Schulz et al., 2021; Wang et al., 2021). The tamponade effect of a hydrogel is exerted by the viscosity and swelling pressure, the latter *via* the high water-binding property; therefore, it seems logical to develop hydrogels based on hyaluronic acid, as this is one of the main components of the natural vitreous body (Nakagawa et al., 1997; Schramm et al., 2012). To further enhance the mechanical stability, chemical cross-linking was investigated as a possible solution (Roy et al., 2012; Spitzer et al., 2012; Schnichels et al., 2017). However, to avoid the risk of potential toxic effects of unreacted cross-linkers, thiolated hyaluronic acid forming disulfide bridges by air oxidation without the need of additional chemical cross-linkers was previously introduced as a promising potential novel vitreous body substitute (Schnichels et al., 2017; Januschowski et al., 2019). These hydrogels showed superior efficacy over silicone oil in a rabbit model of retinal detachment (Schnichels et al., 2017).

Further long-term *in vivo* studies are needed to exclude side effects on surrounding tissue, particular to the retina. Therefore, the aim of the study was to evaluate the long-term biostability and histocompatibility of a highly viscously thiol-modified cross-linked hyaluronate (TCHA) in a rabbit model after vitrectomy.

## Materials and Methods

### Preparation of TCHA Hydrogels

The test substance is a biodegradable, clear, viscous hydrogel, free of visible particles, homogenized and prepared as injectable gel implant, which had been steam sterilized. The steam processing does not affect the physicochemical properties of the hydrogel. Formulations were prepared in physiological phosphate buffer (320 mOsmol/kg, pH 7.4) and contained 2.2% (ViBos strong) HA. Cross-linking was achieved by induced disulfide bridge formation in substituted thiol groups. The rheological analysis and the determination of the refractive index were performed as described in Schnichels et al. (2017).

### Animals and Experimental Setup

All animal procedures and methods were performed in accordance with the Statement for the Use of Animals in Ophthalmic and Vision Research of the Association for Research in Vision and Ophthalmology (ARVO), complied with institutional guidelines and EU and German law, and were conducted under research permission AK1/14, granted by the *Regierungspräsidium* Tübingen, Germany.

All experiments were performed on chinchilla bastard rabbits (ChBB:CH; Charles River Laboratories, Sulzfeld, Germany), after an acclimatization period of at least 5 weeks. Housing and husbandry conditions were compliant with EU guidelines 2010/63/EU. The animals were housed in a pathogen-free facility, one rabbit per cage (Tecniplast, Tecniplast Deutschland GmbH, Hohenpeißenberg, Germany), with straw bedding, allowed free water and food access, and kept in 12-h light/dark cycles, 18°C, and air humidity of 55 ± 10%.

The 18 animals were divided into three subgroups (defined as follow-up 1, 3, or 6 months) of six rabbits each. At the end of the follow-up period, the animals were sacrificed and processed for histology and immunohistology.

The preoperative examinations of the 2-month-old pigmented rabbits included ERG (electroretinography), OCT (optical coherence tomography), and slit-lamp and fundus examination of both eyes and measurement of IOP. Postoperative follow-up examinations are defined in Table 1. ERGs, OCT examination, and surgeries were performed under general anesthesia using intramuscular injections of ketamine 10% (0.25 mg/kg) (Ketanest; Parke Davis, Berlin, Germany) and medetomidine hydrochloride (35 mg/kg) (Sedator; Eurovet, Bladel, Netherlands). Slit-lamp and fundus examination examinations were performed under sedation with 25 mg/kg medetomidine. In addition, local anesthesia with Oxybuprocaine drops (Novesine 0.4%; Novartis, Nürnberg, Germany) was applied throughout all the examinations, IOP measurement, and surgeries. Postoperatively, all animals received 5 mg/kg carprofen to reduce any possible pain.

**TABLE 1:** Examination schedule.

Examination schedule					
	Preoperative examination	Day 1+3+7 post-OP	1 month post-OP	3 months post-OP	6 months post-OP
Weight	X	x	x	x	x
Slit lamp	X	x	x	x	x
IOP OS/OD	X	x	x	x	x
Funduscopy	X	x	x	x	x
ERG	X		x	x	x
OCT	x		x	x	x

### Slit-Lamp and Fundus Examination

Each rabbit was pre- and postoperatively at all follow-up examinations carefully examined on both eyes, specifically to signs of inflammation, fibrin, and small blood clots or signs of incipient cataract with a Kowa SL-15 portable slit lamp.

### Measuring the Intraocular Pressure

IOP of both eyes was measured pre- and postoperatively at all follow-up examinations using a Schiötz tonometer (Winters Tonometer, Germany). The rabbits were placed in the lateral position, and the Schiötz tonometer was launched smoothly perpendicular to the rabbit eye for three times.

### Optical Coherence Tomography

The retina was examined at baseline and at 1, 3, and at 6 months using a high-resolution OCT (SPECTRALIS^®^, Heidelberg Engineering, Heidelberg, Germany). The OCT scanner acquired images with an optical resolution of 7 µm axially and 30 µm laterally and a digital resolution of 3.9 µm axially and 11 µm laterally. The maximum scan depth was 1.9 mm in tissue. The image size was 1,536 × 496 pixels. During each examination, the optic nerve head and visual streak were examined. Thereafter, retinal thickness was determined temporally from the visual streak (volume scan with 25 scans).

### Electroretinography

ERG measurements were performed with an Espion (Diagnosys LLC, Cambridge, UK). After the baseline examination, we scheduled the follow-up examinations after 1, 3, and 6 months postoperatively. 45 min before ERG measurement, one drop of tropicamide 0.5% (Mydriatikum Stulln, Stulln, Germany) was applied for pupil dilation in both eyes. The dark adaptation time period was 30 min. To record the ERG, two contact lens electrodes and three needle electrodes (subcutaneous, one in the right upper lid, one in the left upper lid, and one in the neck) were mounted. To avoid exposure keratopathy, METHOCEL (2% methylcellulose; OmniVision GmbH, Puchheim, Germany) was applied regularly to the cornea. Measurements were performed only when acceptable impedance levels of less than 10 kΩ at 25 Hz (using the machine’s built-in algorithm) were reached. The used dark-adapted and light-adapted ERG protocols, as well as oscillatory potentials, were performed as described in Schnichels et al. (2017).

### Surgery

Pars plana vitrectomy (ppV) was performed under a standard ophthalmic operating microscope (Carl Zeiss Meditec, Inc., Oberkochen, Germany) by one experienced surgeon. In each case, the right eye was operated, and the left eye served as the control. A standard 20/23-gauge vitrectomy system was used (PentaSys 2, Fritz Ruck Ophthalmologische Systeme GmbH, Eschweiler, Germany). Initially, a sclerotomy was placed in the infratemporal quadrant and an infusion line was connected and sutured with 7–0 vicryl (Johnson & Johnson Intl, New Brunswick, NJ, USA). Two further sclerotomies were similarly placed through the pars plana in the two superior quadrants and a light and vitrector inserted into the vitreous. Then, a subtotal vitrectomy without specific posterior vitreous detachment was performed. After vitrectomy with fluid-air exchange, the port was removed and the entire vitreous cavity was filled with ViBos strong using a curved 20-G Weber cannula until egress was witnessed from the second sclerotomy. At the end, the sclerotomies and the conjunctival wounds were closed using 7.0 vicryl absorbable sutures (Ethicon, Norderstedt, Germany). No leaks were witnessed. The operated eye received ointment bid Gentamicin sulfate 5 mg with dexamethasone 0.3 mg (Dexamytrex^®^, Bausch + Lomb, Berlin, Germany) for 1 week to control postoperative inflammation and prevent postoperative infection.

### Histology and Immunohistochemistry

At the end of the examination period, both eyes were removed and were incubated for 5 days in 4.5% formaldehyde. Subsequently, the eyes were first cut in two halves for macroscopical examination and the lens was removed. Afterward, the eye cups were processed for histology and immunohistochemistry with anti-GFAP (glial fibrillary acidic protein) and anti-Brn3a (brain-specific homeobox/POU domain protein 3A) antibodies (Quina et al., 2005). Three representative Brn3a sections (×200) of each case with attached retina were used for the determination of the number of the retinal ganglion cells. The amount of GFAP staining was graded by three independent, blinded examiners (N.S., J.H., S.S.), and an average score for each section was created. The staining was graded as mild (Schulz et al., 2021), moderate (Lin et al., 2021), strong (Kim et al., 2008), or very strong (Duan et al., 2011).

## Results

### Slit-Lamp Examination/Fundoscopy

Rabbits are very prone to noninfectious inflammation after intraocular surgery. Thus, it was not surprising that 13 out of 18 rabbits showed a mild to moderate fibrinous reaction in the anterior chamber on the first postoperative day. A mild amount of fibrin was present in six out of 18 rabbits up to 1 month after surgery. However, after 3 months no fibrin could be detected in any of the animals. There were no signs of infections in any of the rabbits. The cornea stayed clear in all rabbits throughout the entire study.

The formation of cataracts could be observed in five out of 18 animals (28%). Two of these cases were observed in the 1-month group (M1), three in the 3-month group (M3), and none in the 6-month group (M6). All cases with a developed cataract were due to an iatrogenic touch of the posterior lens capsule during the surgery. Thus, all cataracts which developed were most likely iatrogenic due to the surgical procedure and not related to ViBos strong. Also, the type of cataract that developed (posterior capsular cataracts commencing from the area of iatrogenic lens touch) is in agreement with the assumption that the lens opacification was due to technical surgical complication, but not to the hydrogel. Moreover, in the group with the longest follow-up in which no surgical complications occurred, the lenses of all animals stayed clear throughout the entire study.

Four out of the 18 rabbits developed retinal detachments (three in M1 with a follow-up of 1 month; one in M3 with a follow-up of 3 months; none in M6 with a follow-up of 6 months). These cases were more likely to be considered iatrogenic complications. The cases with retinal detachment and flat ERG were excluded from further OCT and ERG analysis. Fundoscopy in all other cases without pronounced cataracts or retinal detachment was unremarkable; especially, no inflammatory reactions at the posterior segment were noticed. During follow-up, the degradation of the hydrogel in the vitreous cavity could not be reliably quantified funduscopically due to the examination conditions and the clear optical properties of the hydrogel.

### Evaluation of Intraocular Pressure

**FIGURE 1:**
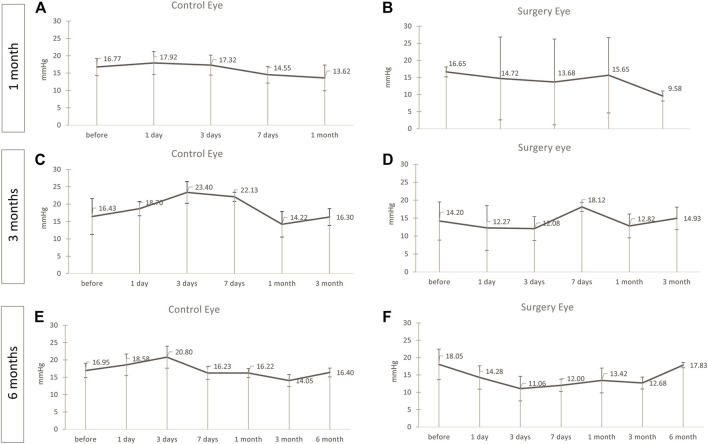
No significant changes in the intraocular pressure (IOP) was noticed. IOP during the follow-up of the operated and contralateral eye after 1 **(A,B)**, 3 **(C,D),** and 6 **(E,F)** months after surgery. The IOP was well within clinically tolerable levels in all groups, but slightly lower in the operated eye than in the contralateral control eye in the 1-month group [**(A,B)**, *p* = .22] and the M3 group [**(C,D)**, *p* = .028]. A similar, but not significant, trend was seen in the M6 group [**(E,F)**, *p* = .058). Points and error bars indicate mean and standard deviation.

After instillation of ViBos, the IOP never reached worrisome high or low levels at any time point after surgery. Although the IOP in the M1 group after 1 month of surgery was slightly lower on average than the contralateral control eye, the IOP was still well within clinically tolerable levels (mean IOP over the entire period: control eye: 16.03 ± 1.8; surgery eye: 14.05 ± 2.7; *p* = .22, Figures 1A,B). The same was detected in the M3 group with a 3-month follow-up (control eye: 18.95 ± 3.8; surgery eye: 14.04 ± 0.2.5; *p* = .028, Figures 1C,D). A similar, but not significant, trend was seen in the M6 group (control eye: 16.74 ± 2.4; surgery eye: 13.26 ± 2.3; *p* = .058, Figures 1E,F).

### Retinal OCT Evaluation

**FIGURE 2:**
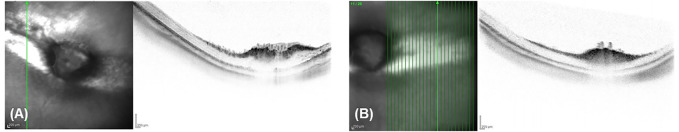
Retinal thickness was not affected. Representative OCT 6 months after surgery showing no difference of integrity, attachment, and thickness of the retina between a treated **(A)** and a control eye **(B)**.

In order to visualize the retinal attachment and the fundus of the eye, OCT examinations were performed in each rabbit pre- and postoperatively. The integrity, attachment, and thickness of the retina were examined during the follow-up of 6 months, and at no time point any difference between treated and control eyes could be observed (Figure 2).

### Scotopic and Photopic ERG

Baselines of all ERG measurement of each group were analyzed for differences between the operated and the control eye. There was no statistically significant difference between the right and left eyee at baseline, and ERG measurements were normally distributed (data not shown).

**FIGURE 3:**
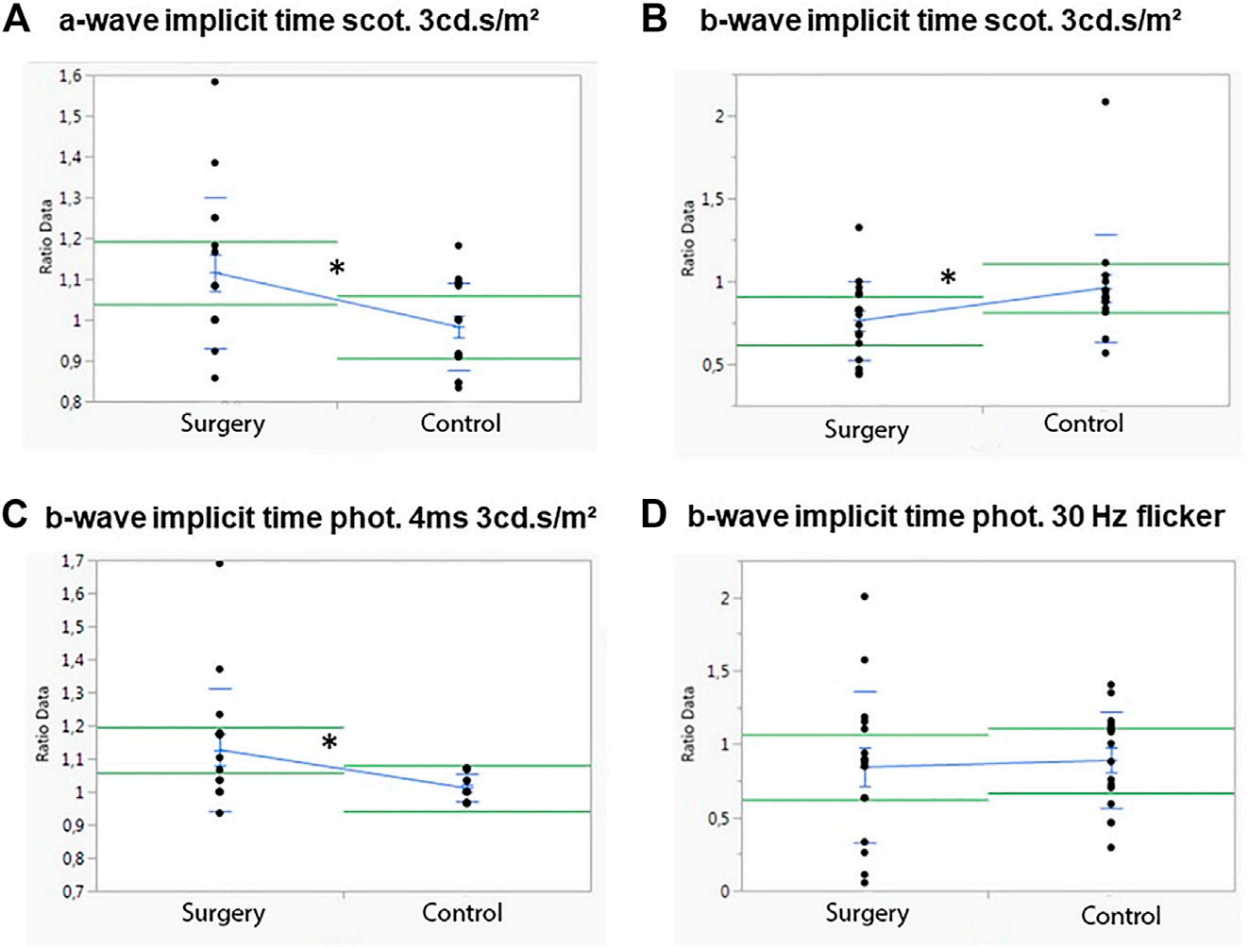
Influence of ViBos strong on the physiological activity of the retina after 1 month. **(A)** A significant higher a-wave implicit time (scotopic standard flash at 3 cd s/m^2^) for the operated compared to the control eyes was measured (*p* = .02). **(B)** Significant differences were detected for the b-wave implicit time (scotopic standard flash at 3 cd s/m^2^) of the control eyes (*p* = .031). **(C)** Significant differences were recorded for the b-wave implicit time (photopic 4 ms at 3 cd s/m^2^) (*p* = .028). **(D)** No significant changes for the b-wave implicit time (30 Hz flicker at 3 cd s/m^2^) were noted (*p* = .769).

In M1, no significant differences between the operated and the control eye were observed for the scotopic a-wave amplitude, but the operated eyes showed a significant higher implicit time than the control eyes (*p* = .02) (Figure 3A). The scotopic b-wave amplitude revealed no differences, but the control eyes had a significant higher b-wave implicit time than the operated eyes (*p* = .031) (Figure 3B).

No differences were measured in the photopic (photopic 4 ms at 3 cd s/m^2^) a-wave amplitude or in the a-wave implicit time after 1 month (data not shown). The same was true for the photopic b-wave amplitude, but a significant increase in the photopic single flash b-wave time (*p* = .028) was observed (Figure 3C). Apart from that, no differences in the control eye were noted for the b-wave implicit time with a 30-Hz flicker at 3 cd s/m^2^, *p* = .769) (Figure 3D).

**FIGURE 4:**
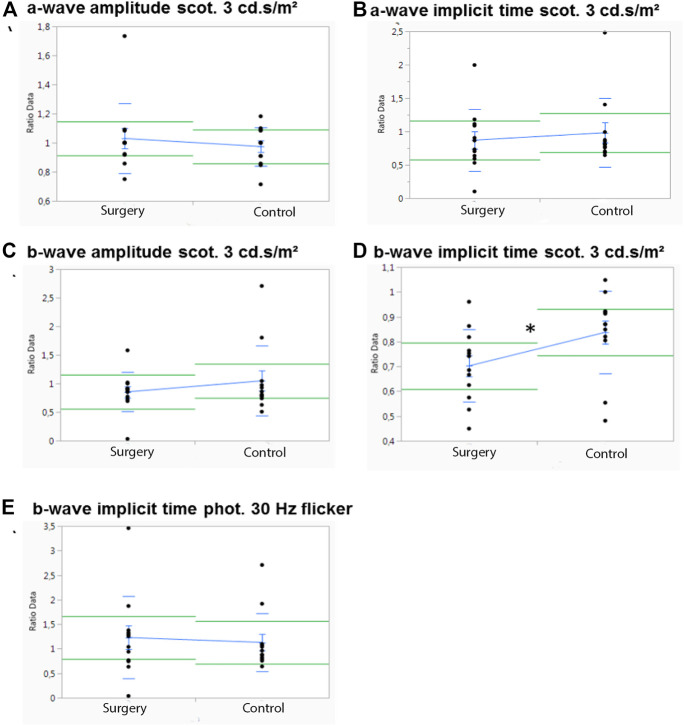
Influence of ViBos strong on the physiological activity of the retina after 3 months. **(A,B)** No significant differences were noted for a-wave amplitudes and a-wave implicit time at 3 cd s/m^2^ (*p* = .58 and *p* = .05, respectively). **(C,D)** There was no effect on the scotopic b-wave amplitudes, but a significant higher b-wave implicit time (*p* = .045) was observed in the control group (scotopic standard flash at 3 cd s/m^2^). **(E)** No significant differences were noted for the b-wave implicit time (30 Hz flicker at 3 cd s/m^2^) (*p* = .742).

In M3 which was followed for 3 months, significant differences between the operated and the control eye were observed only for the scotopic b-wave implicit time (*p* = .045) (Figure 4D). A trend in the scotopic a-wave amplitude, where the control eyes showed a higher a-wave implicit time (*p* = .05), was noted (Figure 4B). Otherwise, no differences in the control eyes were observed in this group (Figures 4A,C,E). There were also no significant differences in photopic a- or b-wave amplitudes or for the implicit time (data not shown).

In M6, no significant differences of the ERG measurements between the operated (gel-filled) and the control eye were observed at any time point (data not shown).

### Macroscopic Examination

**FIGURE 5:**
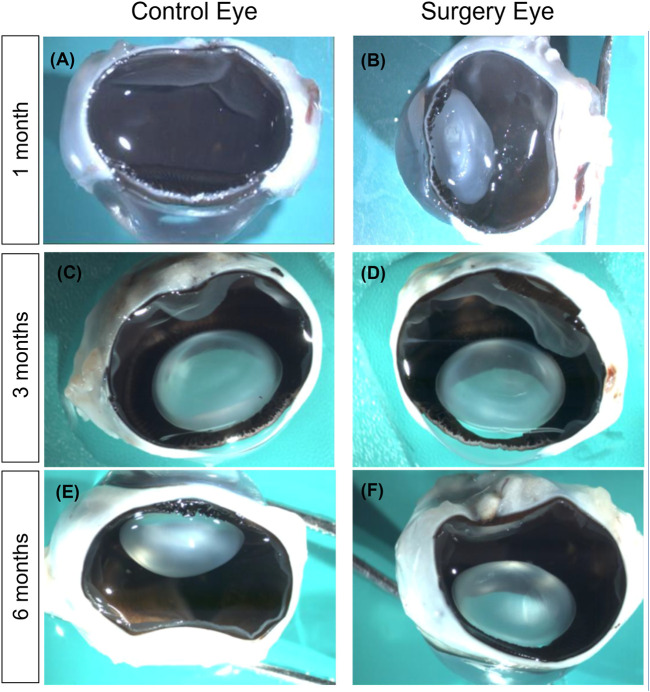
Macroscopic examination of the eyes. **(A,B)** Macroscopic examination after 1 month. The retina of the surgery eye (right) remained completely attached, and the lens was clear apart from a moderate posterior capsular opacification that was due to lens touch during surgery. The vitreous cavity was nearly completely filled with the vitreous substitute. Notably, formaldehyde fixation for histology liquefied the vitreous substitute **(B)**. Macroscopic examination after 3 months **(C,D)**. No differences between treated **(D)** and untreated eyes **(C)** could be observed. However, most of the vitreous substitute had dissolved **(D)**. **(E,F)** Macroscopic examination after 6 months. No differences between treated **(F)** and untreated eyes **(E)**. No vitreous substitute was present at this time point anymore. Magnification ×200.

Macroscopic examination 1 month after implantation of the substitute into the vitreous cavity of the operated eyes showed a complete filling with the tamponade and a partly filled eye after 3 months (Figures 5B,D). The partner eye was always examined as a comparison for possible macroscopically visible changes (Figures 5A,C and E). After 6 months, no gel could be detected by macroscopic examination (Figure 5F). Remarkably, the lens stayed clear throughout the entire follow-up period—except in the cases of iatrogenic lens touch during surgery.

### Histological Examination

**FIGURE 6:**
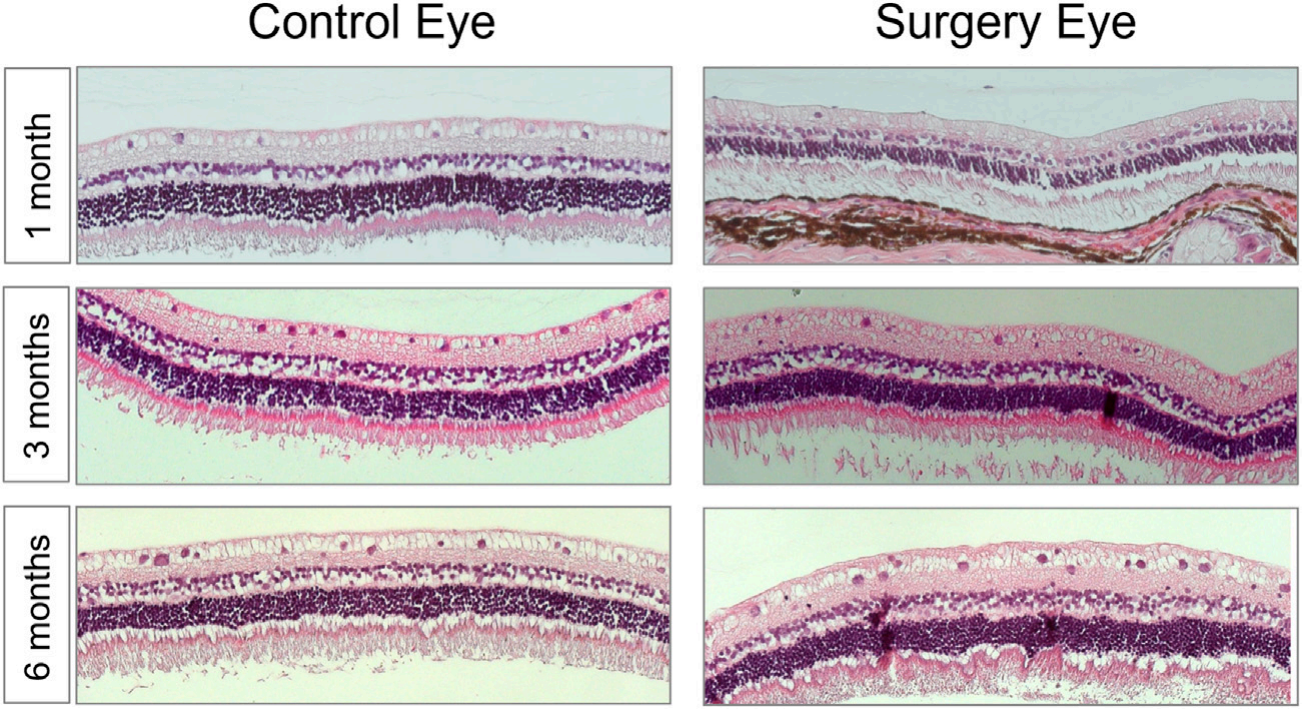
The morphology of the retina was not affected by the vitreous replacement. Representative images of HE-stained retina over the entire time course. 1 month postoperatively; 3 months postoperatively; 6 months postoperatively. No signs of structural damage or toxicity to the retina were detectable. Magnification ×200.

Hematoxylin/eosin (HE) staining was used to examine the retinal structure for its physiological make-up or abnormalities of the structure. In all the eyes examined, only one animal in the 3-month group was found to have high-grade changes in the cell assemblies and a loss of structure of the retinal structure due to retinal detachment. In the remaining seventeen rabbits of the study, no abnormalities of the retinal structure could be detected. In summary, normally structured retinas without any signs of intraretinal or intravitreal inflammation were observable after histological (HE), periodic acid–Schiff (PAS), and immunohistochemistry stainings (Figure 6 and data not shown).

### GFAP and Brn3a Protein Expression (Immunohistochemistry)

**FIGURE 7:**
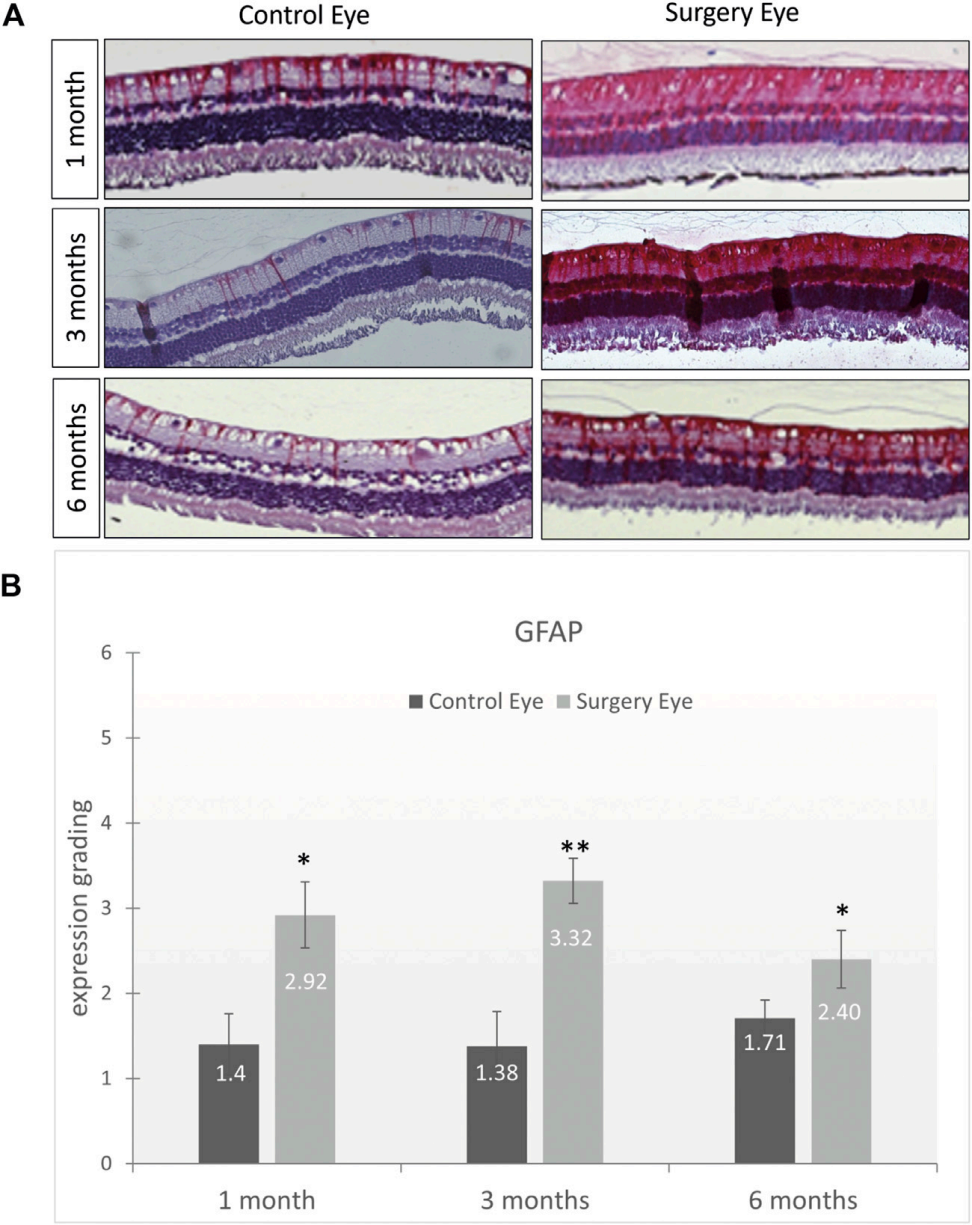
Low activation of macroglial cells by ViBos strong. **(A)** Representative pictures of GFAP staining. **(B)** The GFAP staining was graded as mild (Schulz et al., 2021), moderate (Lin et al., 2021), strong (Kim et al., 2008), or very strong (Duan et al., 2011). Although the GFAP expression was significantly upregulated in all operated eyes throughout the entire study, the total amount of GFAP expression decreased over time. After 1 month, the control eyes got a grading of 1.4 and the surgery eyes 2.92 (*p* = .028; *n* = 3 pairs). The expression of GFAP after 3 months was graded with 1.38 in the control eyes and 3.32 in the surgery eyes (*p* = .0043; *n* = 5 pairs). A slight decrease was observed after 6 months with a grading of 1.71 on the control eyes and 2.3 in the surgery eyes (*p* = .026; *n* = 6 pairs). Bars and error bars indicate mean and standard deviation. Significances are indicated as * with respect to control eyes, using the following significance level: *p < .05; **p < .01.

The expression of *glial fibrillary acidic protein* (GFAP) is an indicator for glial activation and retinal fibrosis. Although the GFAP expression was significantly upregulated in all operated eyes throughout the entire study (1 month: 2.92 vs. 1.2; *p* = .08, 3 months: 3.32 vs. 1.38; *p* = .0043 and 6 months: 2.40 vs. 1.71; *p* = .026), the total amount of GFAP expression decreased over time. The level of upregulation, although statistically significant, is most likely clinically irrelevant as vitrectomy itself causes a significant glial activation and GFAP upregulation. Since the partner eyes that were used for comparison did not receive surgery, the moderate GFAP upregulation in the ViBos strong-filled eyes underlines the good biocompatibility of ViBos strong (Figure 7).

**FIGURE 8:**
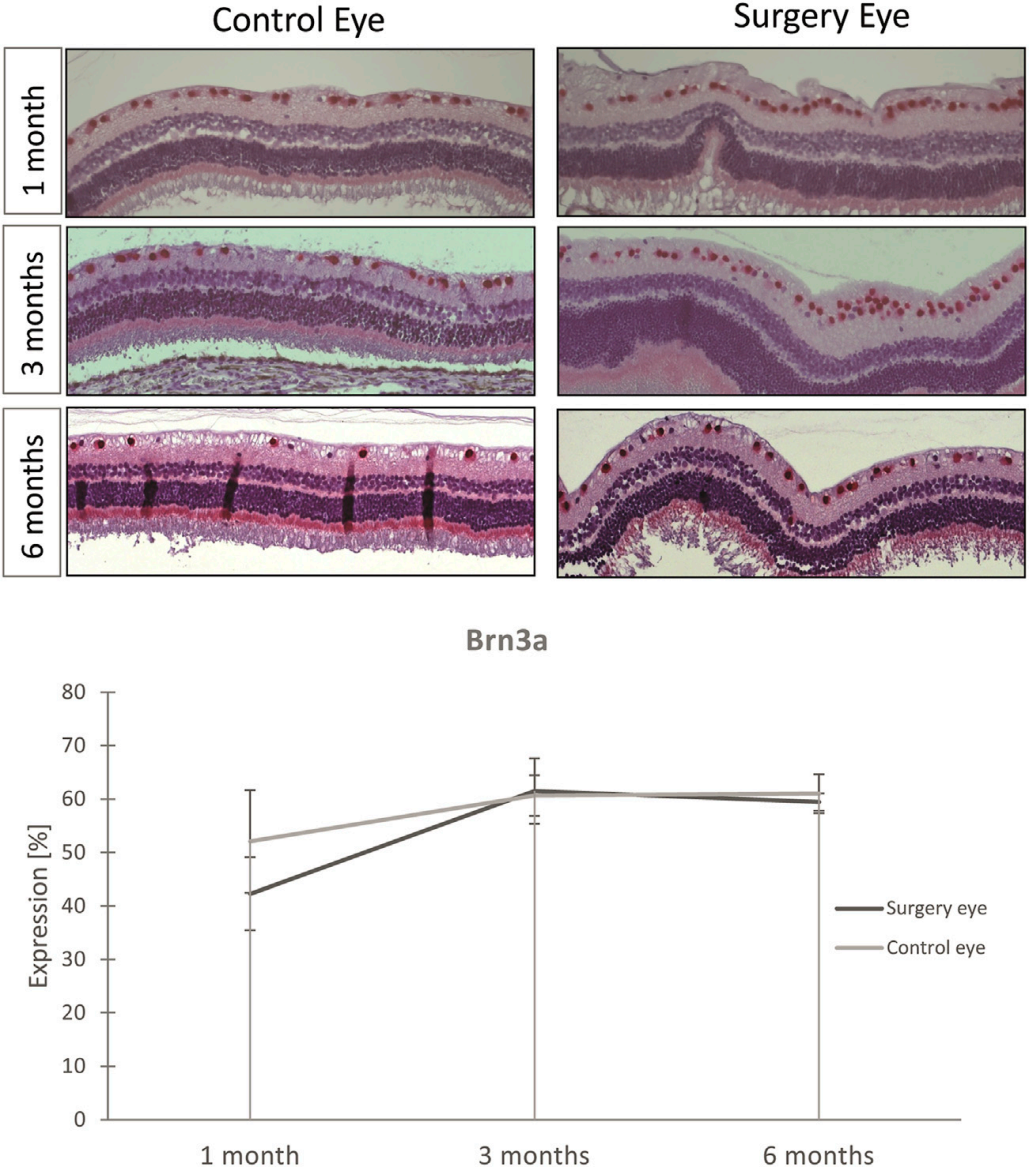
Influence of ViBos strong on retinal ganglion cells. **(A)** Representative pictures of the Brn3a staining. **(B)** Quantitative representation of the relative Brn3a expression. After 1 month, the average percentage of RGC in the ganglion cell layer was 43.27% in the operated eyes and 52.09% in the nonoperated fellow eyes (*p* = .243; *n* = 3 pairs). In the operated eyes of the study over 3 months, it was 61.51% compared to the controls with 60.65% (*n* = 5 pairs). In the 6-month group, the average percentage of RGC in the operated eyes was 59.43% and that of the non-operated fellow eyes 61.02% (n = 6 pairs). Points and error bars indicate mean and standard deviation.

The Brn3a serves as a marker for RGCs. The expression of Brn3a was not significantly affected by vitrectomy with ViBos strong. Thus, the number of RGCs was not diminished after ViBos strong injection (Figure 8). The stable number of Brn3a-positive cells in the ganglion cell layer (GCL) through the whole observation time indicates excellent biocompatibility of ViBos strong and its degradation products even after 6 months. The mean percentage of RGCs in the GCL in the operated eyes of the 1-month group was 43.27% (SD 9.61), and that of the nonoperated partner eyes 52.09% (SD: 6.84). In the operated eyes of the 3-month study, the RGC ratio was 61.51% (SD 3.82) vs. 60.65% (SD: 6.14) of the nonoperated partner eyes. In the 6-month group, the average percentage of RGZ in the operated eyes was 59.43% (SD: 3.62) and 61.02% (SD: 1.65) in the non-operated partner eyes. No significant differences were found between the groups.

## Discussion

Here we presented the result of a long-term *in vivo* study (1, 3, and 6 months) of a highly viscous hydrophilic hydrogel based on thiolated cross-linked hyaluronate (ViBos strong). Good biocompatibility without toxicity or evidence of anatomic or functional changes in a rabbit model was demonstrated in the whole period of 6 months. It could thus be confirmed that ViBos strong is well injectable, transparent, biocompatible, stable over time, and biodegradable (Schnichels et al., 2017).

Despite many years of research, very few polymeric hydrogels can be applied practically in the vitreous cavity yet, due to either a short intravitreal half-life or inflammatory or even toxic effects at the retina and in the vitreous cavity (Nakagawa et al., 1997; Suri and Banerjee, 2006; Wang et al., 2021). Although it seemed obvious to develop VBS based on native hyaluronate (HA), previous studies could only show a very short half-life of less than 14 days (Nakagawa et al., 1997) of non-cross-linked HA in the vitreous cavity and an increase in IOP due to intensive swelling of the hydrogel (Pruett et al., 1979).

To prevent rapid biodegradation, chemically modified and UV cross-linked HA was developed and demonstrated a good biocompatibility, an excellent refractive index of 1.338, and a residence time of the hydrogel in the vitreous cavity of at least 6 weeks to several months, without inflammatory or toxic reaction and no lens opacification (Schramm et al., 2012; Spitzer et al., 2012; Barth et al., 2016; Barth et al., 2019; Gong et al., 2020). However, drawbacks of chemically cross-linked hydrogels are the potential toxicity of the monomers and/or cross-linkers remaining in the eye, and the need for precise control over the injection time (Naahidi et al., 2017; Schulz et al., 2021). Therefore, a stable thiol-modified hydrogel built by natural formation of disulfide bridges by air oxidation without additional chemical cross-linkers (Schnichels et al., 2017) was used for this study. Physicochemical characterization of the naturally cross-linked gel revealed a refractive index similar to the human vitreous, and the transparent, hydrophilic material was shown to be highly elastic but still easily injectable and comparable shear moduli to other vitreous substitutes (Swindle et al., 2008; Santhanam et al., 2016; Schnichels et al., 2017).

Stability and persistence of a vitreous substitute within the vitreous cavity over a longer time period is of great importance, especially in the treatment of complicated retinal detachments where an effective compression of retinal tears according to the orientation of the holes is mandatory. The potential of ViBos strong has already been demonstrated in a retinal detachment model in rabbits by Schnichels et al. in which both the good biocompatibility and the superior efficacy for reattachment of the retina compared to silicone oil could be shown over 1 month (Schnichels et al., 2017). The ViBos is intended to adhere to all locations of the retina due to its swelling properties. This should prevent the occurrence of re-detachment and PVR contralateral to the buoyancy vector, as we see with hydrophobic tamponades and their residual intravitreal fluid. However, this was not explicitly investigated in this study.

Our study with long-term results follows up on these findings and shows that an injection of ViBos strong through a 20-gauge needle was uneventful and showed complete filling of the vitreous cavity with gradual degradation after 3 months. Overall complications were minor with expected fibrin reaction of the anterior chamber after the surgery, and iatrogenic cataract and retinal detachment at the beginning of the experiments, which is in concordance with other studies (Schnichels et al., 2017; Gong et al., 2020). The defined stability of the hydrogel did not lead to a clinically relevant increase of the IOP at any time but had a tendency to a lower IOP. Especially in the first 24 h after vitreoretinal surgery, which are the most critical (Wong et al., 2011) as most of the hydrogel swelling occurs at that time, the IOP was within safe parameters (Januschowski et al., 2019). While the hydrogel degraded slowly over 6 months, there was no evidence of impairment of retinal function from potential degradation products in the OCT and histological examinations. In accordance, retinal function was well preserved after the instillation of the hydrogel. Some minor impairments of the ERGs 1 or 3 months postoperatively were most likely due to the surgical procedure and not due to the filling with ViBos strong. This assumption is strongly supported by the normal ERG findings 6 months after surgery. Moreover, it was most remarkable that after 6 months of follow-up the ERG of the eyes that underwent surgery was not significantly different from the ERG of the contralateral control eye, given the fact that vitrectomy on its own can cause some decrease of retinal function. A similar observation with normalization of ERG function after vitreoretinal surgery was also shown in another model (Liu et al., 2019). Whether the hydrogel itself also has an influence on ERG accuracy has not been conclusively clarified to date. However, this seems very unlikely as the hydrogels unlike the hydrophobic silicone oil mainly consist of water. The water content of thy hydrogels is very similar to that of the natural vitreous.

In conclusion, the tested hydrogel had a very good tolerance without toxic properties on the retina. The good optical properties of the hyaluronic acid-based hydrogel, which almost correspond to the physiological vitreous body, could also enable good visual properties of the patients postoperatively. Therefore, the proven biocompatibility of ViBos strong over 6 months is significant for the application in the clinical field and might be a promising agent for further studies of retinal tamponades.

## Data Availability

The raw data supporting the conclusion of this article will be made available by the authors, without undue reservation.
